# Iron-biofortified pearl millet consumption increases physical activity in Indian adolescent schoolchildren after a 6-month randomised feeding trial

**DOI:** 10.1017/S000711452100180X

**Published:** 2022-04-14

**Authors:** Laura M. Pompano, Sarah V. Luna, Shobha A. Udipi, Padmini S. Ghugre, Eric M. Przybyszewski, Jere Haas

**Affiliations:** 1 Cornell University, Savage Hall, Ithaca, NY 14853, USA; 2 S.N.D.T. Women’s University, 1, Nathibai Thackersey Road, Mumbai 400020, India; 3 HarvestPlus, International Food Policy Research Institute, 1201 I St NW, Washington, DC 20005 USA

**Keywords:** Adolescent health, Iron deficiency, Physical activity, Accelerometry, Iron biofortification, Pearl millet, Sedentary behaviour

## Abstract

Fe deficiency has negative effects on voluntary physical activity (PA); however, the impact of consuming Fe-biofortified staple foods on voluntary PA remains unclear. This study compared the effects of consuming Fe-biofortified pearl millet or a conventional pearl millet on measures of voluntary PA in Indian schoolchildren (ages 12–16 years) during a 6-month randomised controlled feeding trial. PA data were collected from 130 children using Actigraph GT3X accelerometers for 6 d at baseline and endline. Minutes spent in light and in moderate-to-vigorous PA were calculated from accelerometer counts using Crouter’s refined two-regression model for children. Mixed regression models adjusting for covariates were used to assess relationships between intervention treatment or change in Fe status and PA. Children who consumed Fe-biofortified pearl millet performed 22·3 (95 % CI 1·8, 42·8, *P* = 0·034) more minutes of light PA each day compared with conventional pearl millet. There was no effect of treatment on moderate-to-vigorous PA. The amount of Fe consumed from pearl millet was related to minutes spent in light PA (estimate 3·4 min/mg Fe (95 % CI 0·3, 6·5, *P* = 0·031)) and inversely related to daily sedentary minutes (estimate −5·4 min/mg Fe (95 % CI –9·9, −0·9, *P* = 0·020)). Consuming Fe-biofortified pearl millet increased light PA and decreased sedentary time in Indian schoolchildren in a dose-dependent manner.

Fe deficiency is the most common micronutrient deficiency in both developed and developing countries^(1,2)^. Previous literature has shown that improving the Fe status of Fe-deficient adults can increase their physical activity (PA) levels, with both factory workers and tea pickers showing improvements in their daily PA after receiving Fe supplements^(3,4)^. Additionally, Crouter *et al.* reported that women with healthy Fe levels performed 52·1 more min/d of light PA (LPA) and spent 68·4 fewer min/d in sedentary behaviours compared with women with poor Fe status^(5)^. These studies collectively suggest that resolving Fe deficiency in adults increases LPA – which has been shown to be beneficially associated with mortality, lipid and glucose metabolism, and obesity^(6)^ – and may also reduce sedentary behaviour, potentially as a result of Fe-deficiency-related fatigue^(7)^.

While interventions such as food fortification, dietary Fe supplementation or increasing dietary diversity are all effective strategies for addressing Fe deficiencies in many populations, these methods are often not feasible or accessible for the rural poor and other high-risk populations^(8)^. One method developed to reach these groups is biofortification, or the process of increasing the concentration and bioavailability of essential micronutrients in staple crops using conventional plant breeding and agronomic practices. The technique has the potential to become a sustainable, inexpensive and effective solution to Fe deficiency at the population level^(9)^. Pearl millet (*Pennisetum glaucum*) is a staple food in India, particularly in the states of Rajasthan, Gujarat and Maharashtra^(10,11)^, where this study took place. Previous work by our research group has demonstrated that the consumption of Fe-biofortified pearl millet was able to improve the Fe status and cognitive function of Indian children^(12,13)^. However, the effects of consuming an Fe-biofortified staple food on voluntary PA, which is known to be related to Fe deficiency^(14–16)^, remain unclear.

While previous studies have focused on Fe-deficient adults, it is logical that a similar relationship could be observed in children; however, to date, the relationship between Fe status and voluntary PA in children remains poorly understood^(12)^. Therefore, the objective of this study was to examine the effect of consuming Fe-biofortified pearl millet during a 6-month randomised controlled feeding trial on objective measures of voluntary PA (time spent in light, moderate or vigorous PA as well as sedentary behaviours) in Indian schoolchildren aged 12–16 years. We hypothesised that participants who consumed Fe-biofortified pearl millet would increase their time spent performing non-sedentary PA compared with those who consumed standard variety pearl millet.

## Methods

### Study participants

**Fig. 1.**
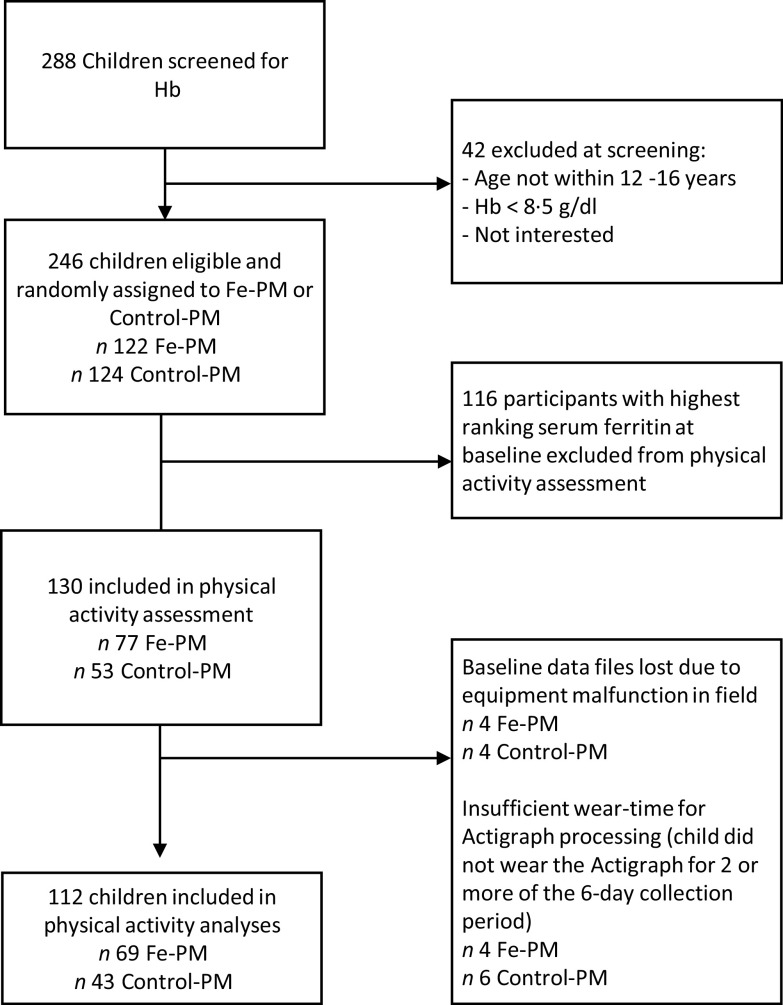
CONSORT diagram of study participant selection. Fe-PM, treatment arm receiving iron-biofortified pearl millet; Control-PM, treatment arm receiving standard variety pearl millet; PA, physical activity.

This study was conducted from September 2011 to March 2012 among schoolchildren (12–16 years) in the Ahmednagar district, Maharashtra, India. Children were not eligible to participate if they: had severe anaemia (Hemoglobin, Hb < 8·5 g/dl), were taking Fe supplements or any medication that could interfere with Fe absorption, had a chronic illness, if they or their parents or guardians were not willing to participate in the study or if they did not reside full time at the boarding school. All children were given anthelmintic treatments 4 weeks prior to the baseline blood collection and again at the study midpoint. Sample selection for the larger parent feeding trial was described in greater detail previously, but is summarised here^(12)^. Two hundred and eighty-eight male and female students were screened for inclusion in the parent feeding study. A total of 246 children were included in the parent analysis comparing the effect of consuming Fe-biofortified pearl millet (hereafter called ‘Fe-PM’) with consuming a popular commercial variety of pearl millet (hereafter called ‘Control-PM’) on changes in Fe status. Because it was not logistically possible to monitor PA for the entire sample of 246 subjects, a subsample of students with worst Fe status was selected to participate in the PA assessment by selecting the 130 participants with the lowest screening serum ferritin (sFer) values in order to understand the relationship between Fe status and PA among those with the greatest potential to benefit from additional dietary Fe. The flow of participants in the present analyses is shown in Fig. 1.

The study participants came from low-income households, lived at one of three hostels on the school campus and took all meals in the communal dining area of each hostel. Participants were selected because they represented an age and socio-economic segment of the Indian population at high risk for Fe deficiency^(17)^ and because their staple cereal was pearl millet. They regularly consumed large quantities of pearl millet (about 150–350 g/d, dried) in the form of *bhakri*, a type of round flatbread, during both lunch and dinner. Daily *bhakri* consumption was monitored and recorded for each subject by trained research assistants. All meals at each hostel were prepared in common kitchens. Dietary intake was assessed at baseline and endline using a 24-h recall administered by a trained research assistant. A food composition database was developed specifically for the diet of the children and was analysed using CS Dietary System software (CS Dietary System, version 1.1). The Fe content of *bhakri* was determined using ICP analyses of random *bhakri* samples collected from the study site every 2 weeks. Total Fe consumption was determined by multiplying the Fe per *bhakri* by the number of *bhakri* consumed. Details of the dietary intake protocol and analysis have been reported previously^(12)^.

### Study design

The two treatments of pearl millet were randomly assigned at the individual level. The pearl millet flour was incorporated into the *bhakri* following a standardised local recipe. The *bhakri* made from Control-PM was identical in colour, taste and phytochemical composition and content as the *bhakri* from Fe-PM. Children were organised into feeding groups at mealtime (lunch and dinner) based on the type of *bhakri* they were to receive. There were two coded groups: one for Fe-PM and one for Control-PM. The distribution of *bhakri* was monitored by assistants who did not know which *bhakri* was assigned to each coded group. Each child’s *bhakri* consumption (by quarters of a whole *bhakri*) was recorded by the assistants. Details of the parent study can be found in Finkelstein *et al.* ^(12)^ The amount of Fe consumed from pearl millet increased for both the Fe-PM and Control-PM groups during the final 2 months (months 4–6) of the trial due to (1) a change in the variety of pearl millet fed to the Control-PM group and (2) the introduction of a pearl millet-based *shev* snack to all children. The Control-PM contained 21·8 parts per million Fe, or 21·8 mg Fe/kg pearl millet, from months 1 to 4 (variety DG9444), then increased to 52·1 parts per million in months 4–6 (variety JKBH778). The Fe-PM contained 86·3 parts per million Fe for the full 6-month intervention (variety ICTP8203). Children were given about 200–300 g of dry pearl millet per day through the *bhakri*, which was provided *ad libitum*.

### Physical activity protocol

The present study’s primary outcome was PA, which was assessed by accelerometers at baseline and after 6 months. PA data were collected in three sets of 40–45 subjects over 3 weeks at baseline and endline. The data were recorded in 10-s epochs using triaxial Actigraph GT3X accelerometers for six consecutive days from Tuesday through Sunday. No data were collected on Mondays, which were reserved for collecting the accelerometers, downloading data and recharging the batteries. Mondays were selected as the ‘off day’ of the week because they were similar to the other 4 school days in the week, when activities were likely to be similar. Movement was recorded in the transverse, frontal and sagittal planes. Trained research technicians placed the accelerometer affixed to an elastic belt worn over the child’s left hip. Each child was instructed to wear the accelerometer at all times, except when sleeping or bathing. The raw count data were assessed for wear-time, with non-wear-time being determined using the Choi algorithm^(18)^. Insufficient wear-time was defined as the child not wearing the device for 2 or more days out of the 6-d collection period, with a valid day being defined as having a minimum of 10 h of wear-time. Metabolic equivalents were calculated from the accelerometer counts using Crouter’s refined two-regression model for children with the R statistical package obtained from the Crouter research laboratory^(19,20)^. PA outcome variables included steps taken and number of minutes spent in sedentary behaviours like sitting in class (metabolic equivalent = 1), light activity like walking, and performing chores or games (LPA, metabolic equivalent 1–3) and moderate-and-vigorous activity like running or playing sports (metabolic equivalent > 3).

### Laboratory analyses

Blood draws were performed at baseline, after 4 months and at 6 months (endline) and assessed for the following blood biomarkers: Hb, sFer and soluble transferrin receptor as well as calculated total body Fe. Fe deficiency was defined as sFer < 15 µg/l. Anaemia was defined as Hb < 12·0 g/dl. Total body Fe was calculated as a ratio of soluble transferrin receptor:sFer using the formula reported by Cook^(21)^. All Fe biomarkers, *α*-1-acid glycoprotein and C-reactive protein were analysed by Metropolis HealthCare Laboratory in Mumbai. Laboratory methods for blood analysis of Fe and inflammation biomarkers were described in detail previously^(12)^.

sFer was adjusted for inflammation using the regression correction approach described by the Biomarkers Reflecting Inflammation and Nutritional Determinants of Anemia project^(22)^. For comparison, all analyses were also run without the Biomarkers Reflecting Inflammation and Nutritional Determinants of Anemia correction, both including and excluding participants with C-reactive protein > 3 mg/l and *α*-1-acid glycoprotein > 1 g/l. No differences were observed in significance levels or the direction or magnitude of treatment effects between the corrected and uncorrected data. All ferritin values presented in the results and tables reflect the Biomarkers Reflecting Inflammation and Nutritional Determinants of Anemia-corrected values and do not exclude individuals with inflammation.

Anthropometry was performed at baseline and 6 months by trained research assistants as previously described^(23)^.

### Statistical analyses

A sample size calculation, based on a previous study examining PA in Fe-deficient and Fe-replete populations^(5)^, showed that a sample size of 53/group would be sufficient to detect a 50·7 min/d difference in LPA between groups assuming an *α* of 0·05 and 90 % power. Descriptive statistics are expressed as means with 95 % CI. One-way ANOVA was used to compare demographic and background characteristics between treatment groups. Tukey adjustments for multiple comparisons were used in ANOVA models with more than two groups. Linear regression models were used to analyse the treatment effects on endline Hb, ferritin, soluble transferrin receptor, body Fe and PA variables. All models included baseline value and sex as covariates. Actigraph wear-time was included as a covariate in all analytical models examining PA outcomes, as recommended by Crouter *et al.* ^(19,20)^. Due to unforeseen problems in the study implementation, there were some limitations that may have reduced our ability to observe effects in the intention-to-treat analyses since most participants were consuming sufficient Fe by month 6. Therefore, several secondary analyses were conducted to explore the biological plausibility of the results observed for treatment group differences. Secondary analyses included testing for changes in Fe status as a predictor of changes in PA using linear regression models, adjusting for the same covariates included in the analyses of intervention effects. An adjusted *P*-value of <0·05 was considered statistically significant for main effects, and a *P*-value of <0·10 was considered statistically significant for interactions. No biomarker-by-sex interactions were observed in any secondary analysis model. All results of secondary analyses are presented with sexes combined, with sex included as a covariate. All analyses were done in SAS 9.4 software (SAS Institute Inc., Cary, NC, USA).

### Ethics

This study was conducted according to the guidelines laid down in the Declaration of Helsinki, and all procedures involving human subjects/patients were approved by the Intersystem Biomedica Ethics Committee in Mumbai, India as well as the Institutional Review Boards of Cornell University, The University of Oklahoma and The Pennsylvania State University. Informed written consent was obtained from each participant as well as their guardians and the management and heads of the schools. This trial was registered at ClinicalTrials.gov as NCT02152150.

## Results

Demographic information is shown in Table 1. There was no difference in any baseline measure between the Fe-PM and Control-PM groups. The distribution of males and females in each group was not statistically different (*χ* ^2^ *P*-value = 0·49).

**Table 1.** Participant characteristics at baseline (Mean values and standard deviations)

	Control-PM		Fe-PM	
	Mean	sd	Mean	sd
*n*	43		69	
Sex (% male)				
%	55·8		62·3	
Age (years)	13·8	1·4	13·6	1·4
Hb (g/dl)	12·4	1·0	12·5	0·9
Ferritin (μg/l)	15·1	8·7	18·0	10·8
sTfR (mg/l)	6·6	2·3	6·8	2·4
Body Fe (mg/kg)	1·3	2·6	1·9	2·8
AGP (mg/ml)	0·6	0·2	0·6	0·2
CRP (mg/l)	0·4	0·7	1·0	3·4
LPA (min at 1–3 MET)	251	56	237	52
MVPA (min at >3 MET)	95	47	96	48
Sedentary time (min)	480	95	494	98

Fe-PM, Fe-biofortified pearl millet; Control-PM, control pearl millet; sTfR, soluble transferrin receptor; AGP, *α*-1-acid glycoprotein; CRP, C-reactive protein; LPA, light physical activity, defined as minutes spent performing activities in the 1–3 metabolic equivalents range; MVPA, moderate-to-vigorous physical activity, defined as minutes spend performing activities above 3 metabolic equivalents.

At baseline, 53·6 % (*n* 60) of the PA sample participants were Fe-deficient (sFer < 15 µg/l) and 34·5 % were anaemic (*n* 41, Hb < 12·0 g/dl), of whom 58·5 % (*n* 24) were Fe-deficient anaemic. Fourteen participants (11·8 %) had soluble transferrin receptor concentrations above 8·3 mg/l, while twenty-nine children (24·4 %) had negative body Fe values. Additionally, 5·4 % (*n* 6) and 4·5 % (*n* 5) of participants had inflammation based on *α*-1-acid glycoprotein (>1 g/l) or C-reactive protein (>3·0 mg/l), respectively. There were no differences in the prevalence of inflammation by treatment group or sex at baseline.

The Control-PM group consumed 5·9 (sd 1·1) mg Fe/d and 9·7 (sd 1·1) mg Fe/d from the pearl millet during months 1–4 and 5–6, respectively. The Fe-PM group consumed 10·7 (sd 2·3) mg Fe/d and 16·5 mg Fe/d in months 1–4 and 5–6, respectively. The Fe-PM group consumed 4·5 mg/d more Fe than the Control-PM group from months 1 to 4 (one-way ANOVA *P* value < 0·001) and 5·6 mg/d more Fe than the Control-PM group from months 5 to 6 (one-way ANOVA *P* value < 0·001). The number of bhakri consumed each day did not differ between the treatment groups, with the Control-PM group consuming 1·1 (sd 0·3) bhakri/d and the Fe-PM group consuming 1·2 (sd 0·2) bhakri per day (one-way ANOVA *P* value = 0·40).

### Effect of treatment on iron status

No effects of treatment on Fe status were observed at 6 months in the present study (*n* 112), but the Fe-PM group had significantly higher body Fe than the Control-PM group at 4 months, on the basis of linear mixed models, controlling for baseline Fe status and sex (Table 2). There were no significant changes in any Fe status measure between 4 and 6 months for either treatment group.

**Table 2.** Iron status at months 4 and 6, adjusting for baseline status and sex* (Mean values and 95 % confidence intervals)

			Control-PM		Fe-PM		Diff (Control-Fe)		
Time point	Outcome	*n*	Mean	95 % CI	Mean	95 % CI	Mean	95 % CI	Treatment *P* value
Month 4	Hb (g/dl)	102	13·8	12·2, 15·4	13·8	12·2, 15·3	–0·1	–0·4, 0·2	0·96
	sFer (µg/l)	91	12·4	5·3, 19·6	16·3	11·3, 21·3	–3·9	–12·7, 5·0	0·39
	Log sFer	91	2·3	2·0, 2·7	2·4	2·1, 2·7	–0·1	–0·4, 0·2	0·52
	sTfR (mg/l)	91	7·7	6·6, 8·8	7·2	6·3, 8·2	0·5	–0·4, 1·3	0·31
	BI	91	1·3	0·6, 2·0	2·3	1·7, 2·9	–1·0	–1·9, −0·1	0·036
Month 6	Hb (g/dl)	98	12·4	12·1, 12·7	12·5	12·2, 12·7	–0·1	–0·4, 0·3	0·75
	Fer (µg/l)	99	19·6	14·6, 24·5	20·1	15·7, 24·6	–0·5	–4·6, 3·6	0·79
	Log Fer	99	2·7	2·4, 3·1	2·8	2·5, 3·1	–0·1	–0·2, 0·1	0·60
	sTfR (mg/l)	99	7·1	6·2, 8·1	7·1	6·3, 8·0	–0·0	–0·7, 0·7	0·93
	BI	99	2·2	1·2, 3·2	2·5	1·6, 3·4	–0·3	–1·1, 0·6	0·51

Control-PM, group receiving control pearl millet; Fe-PM, group receiving Fe-biofortified pearl millet; Diff, difference in LS means for Control-PM and Fe-PM groups; sFer, serum ferritin; sTfR, soluble transferrin receptor; BI, body Fe.

* Results of linear mixed models adjusted for baseline value of outcome and sex with a random effect of hostel (location).

### Effect of treatment on physical activity

Table 3 shows the results of the analyses of the two dietary treatments on PA outcomes at 6 months. No treatment-by-sex interactions were observed for any intention-to-treat model; therefore, results are presented with both sexes combined; however, sex was retained as a covariate in all models. There were no differences in wear-time between treatment groups at baseline (Fe-PM: 827 (sd 79) min, Control-PM: 825 (sd 108) min, one-way ANOVA *P* = 0·91) or at month 6 (Fe-PM: 823 (sd 104) min, Control-PM: 811 (sd 125) min, one-way ANOVA *P* = 0·59).

**Table 3.** Analysis of treatment group differences for physical activity at 6 months* (Mean values and 95 % confidence intervals)

		Control-PM		Fe-PM		Diff (Ctl − Fe)		
Daily physical activity	*n*	Mean	95 % CI	Mean	95 % CI	Mean	95 % CI	TRT *P* value
Sedentary time (min)	112	499	475, 523	475	455, 494	24·4	–6·6, 55·3	0·12
LPA (min)	112	238	212, 263	260	237, 283	–22·3	–42·8, −1·8	0·034
MVPA (min)	112	90	78, 103	88	78, 98	1·9	–14·2, 17·9	0·82
Steps	112	9951	8956, 10 947	9909	9086, 10 731	42	–1110, 1196	0·94

Control-PM, group receiving control pearl millet; Fe-PM, group receiving Fe-biofortified pearl millet; Diff (Ctl − Fe), difference in LS means between Control-PM and Fe-PM groups; TRT *P* value, *P* value for treatment group; LPA, light physical activity, defined as minutes spent performing activities in the 1–3 metabolic equivalents range (METS); MVPA, moderate-to-vigorous physical activity, defined as minutes spend performing activities above 3 METS.

* Results of linear mixed models adjusted for baseline value of outcome, sex, as well as baseline and endline wear-time. All models include a random effect of hostel (location) and are adjusted for multiple comparisons using a Tukey adjustment.

There was a significant effect of treatment on minutes spent in LPA, with the Fe-PM group performing 22·3 min/d (95 % CI 1·8, 42·8 min/d) more LPA than the Control-PM group (linear mixed model *P* = 0·034). No treatment effects were observed for minutes per day in sedentary behaviour, moderate-and-vigorous activity or steps.

### Secondary analyses

First, we examined whether the daily average amount of Fe consumed from pearl millet during the 6-month study differed by treatment group. The treatment group significantly predicted the amount of Fe consumed per day. Participants in the Control-PM group consumed an average 7·3 mg/d (95 % CI 5·8, 8·7 mg/d) over the 6-month trial, while participants in the Fe-PM group consumed an average of 12·8 mg/d (95 % CI 11·4, 14·2 mg/d), *P* < 0·001.

**Fig. 2.**
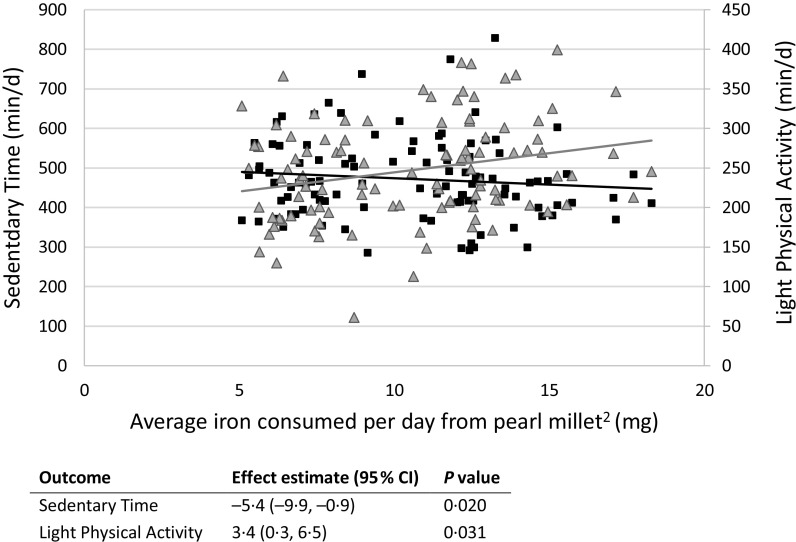
Minutes spent in light physical activity and sedentary behaviour predicted by average iron consumption^1^. ^1^ Results of linear mixed models adjusted for baseline value of outcome, sex and (for physical activity variables) baseline and endline wear-time. All models include a random effect of hostel (location) and are adjusted for multiple comparisons using a Tukey adjustment. ^2^ Refers to the amount of iron consumed from pearl millet over the 6-month study duration and not iron from other dietary sources. 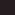, Sedentary; 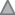, Light Physical Activity.

Second, we examined whether the average amount of Fe consumed per day over the study duration was related to endline PA, adjusting for baseline, sex and wear-time. Participants who consumed more Fe during the study engaged in a greater number of minutes of voluntary LPA and had fewer sedentary minutes, regardless of treatment group (Fig. 2). We also examined whether baseline to endline changes in Fe biomarker concentrations were related to endline PA (adjusting for baseline performance). No significant relationships were observed between changes in any Fe biomarker and any PA outcome (data not shown).

In order to examine the internal consistency of the PA data, we also examined whether the change in LPA minutes was related to change in sedentary time. While treatment group did not impact this relationship, there was a strong inverse relationship between the change in minutes spent performing LPA and minutes spent in sedentary behaviours (effect estimate and 95 % CI −1·3 min (–1·4, −1·1), *P* < 0·001, *R* ^2^ = 0·85).

Finally, we examined the effect of baseline Fe status (Fe-deficient non-anaemic, Fe-deficient anaemia, Fe replete non-anaemic, Fe replete anaemic) on all PA outcome variables. There were no significant differences in any outcome measure related to the Fe status groups at baseline on the basis of one-way ANOVA adjusted for pairwise comparisons using a Tukey correction (data not shown).

## Discussion

The goal of the present study was to determine the effects of consuming Fe-biofortified pearl millet on voluntary PA. The study showed that children who consumed Fe-biofortified pearl millet performed 22·3 min more LPA per day than those who consumed control pearl millet. Specifically, this LPA appears to replace sedentary time, as evidenced by the roughly equivalent, though not statistically significant, decrease in sedentary time in the Fe-PM group. The effect of the intervention was further supported by the relationship between the amount of Fe consumed from pearl millet during the intervention and the changes in sedentary and LPA behaviours, with greater Fe intake being related to more minutes in LPA and fewer sedentary minutes.

This is the first study to show that consumption of an Fe-biofortified staple food increases LPA compared with the consumption of a conventional variety of the staple. This is also the first study to examine PA outcomes in a biofortification intervention in children. The current findings are supported by those of previous research, which have shown that adults with poorer Fe status had increased sedentary time compared with those with normal Fe levels^(5)^. Another study found that adult women who consumed Fe supplements performed 30 min more daily non-sedentary PA than those supplemented with placebo^(4)^, which is similar to the 22 min additional LPA observed in the present study.

Furthermore, the number of minutes children spent in sedentary behaviours was strongly and inversely related to the number of minutes they spent performing LPA, suggesting that consumption of Fe-biofortified pearl millet leads children to replace roughly 22 min of sedentary time with LPA per day. This finding is further supported by our secondary analyses, which showed that the amount of Fe consumed from pearl millet, regardless of treatment group, was directly related to LPA and inversely related to sedentary time in a dose-dependent manner. Specifically, for each additional 1 mg/d of Fe consumed from pearl millet, participants performed 5 fewer min of sedentary activity and 3 additional min of LPA each day.

The dose-dependent response observed in the present study further supports the use of biofortification as a tool to address Fe deficiency in areas with high prevalence of Fe deficiency that are not served by Fe interventions such as supplementation or fortification. While it would be ideal to provide the full recommended daily Fe requirement in an Fe intervention where possible, the results of the present study suggest that replacing conventional pearl millet varieties with Fe-biofortified varieties could impact children’s PA behaviours even if they provide only a portion of the recommended daily intake.

While the physiological mechanism between the consumption of Fe-biofortified crops and PA behaviours remains somewhat unclear, previous research suggests that fatigue may be a contributing factor. A recent meta-analysis showed that Fe supplementation improved self-reported fatigue in women with Fe deficiency without anaemia^(7)^. Similarly, a review by McClung & Murray-Kolb also cite fatigue as an explanation for the observed relationship between Fe status and PA observed in previous studies^(4,5,16)^. Further research should directly examine the results of Fe biofortification interventions on measures of fatigue to explore the mediating role it may have on voluntary PA.

Increased PA (and reduced sedentary time) has been shown to contribute to improved cognitive performance^(16,24)^, executive function^(25)^, academic achievement and motor skills in children^(24,26)^. Additionally, a recent systematic review found that while there is currently a lack of high-quality data specifically looking at the health effects of LPA, existing studies have shown that increased LPA was favourably associated with diastolic blood pressure, insulin resistance and HDL-cholesterol in children and adolescents^(27)^. Another review using isotemporal substitution models found that reallocation of even 30 min of daily sedentary behaviour to LPA or more intense PA was associated with reduced risk of mortality and other health benefits^(28)^. Therefore, increasing daily PA and reducing sedentary behaviours through the consumption of biofortified pearl millet could not only improve the overall activity level of this population but could also lead to healthier development in other key aspects of child growth. Indeed, cognitive function was also shown to improve with the consumption of Fe-biofortified pearl millet in this same study population^(13)^.

This study had several limitations. The first is that the intervention effects on Fe status occurred at 4 months, while PA was only assessed at 6 months for logistic reasons. At 4 months, the Control-PM group was switched to a pearl millet variety with a higher Fe content and both groups received a pearl millet-based snack. These changes resulted in increased dietary Fe intake leading to improvements in Fe status in the Control-PM group between months 4 and 6^(12)^. Without PA measures at 4 months, it was not possible to determine if there was a significant intervention effect on PA at month 4. We considered the possibility that the PA response at 6 months lagged after adequate Fe status was achieved at 4 months. However, there were no significant relationships that supported an effect. In addition, we did not directly measure fractional absorption of Fe from pearl millet. Rather, we assumed a fractional absorption of Fe of 7·5 % from pearl millet based on a previous study^(29)^. Given the ICP analyses and the total number of *bhakri* consumed, the absorbed Fe values at endline were estimated to be approximately 64 % and 109 % of the USA estimated average requirement for this age group and weight (15·1 mg/d for adolescents 12–19 years of age), in the Control-PM and Fe-PM groups, respectively^(12,30)^. We also cannot exclude the possibility that there were differences in other nutrients between the control and high-Fe pearl millet that may have contributed to the results. Despite these limitations, a significant treatment effect was still observed for LPA at 6 months.

Additionally, the sample size of the PA subgroup was smaller than intended (Fig. 1). This was a result of eight lost data files (4 Fe-PM, 4 Control-PM), during transfer of data from the field site. These data are considered to be ‘missing completely at random’ and are not expected to introduce bias into the analyses. Data from another ten subjects (4 Fe-PM, 6 Control-PM) were lost at the analysis phase because they did not wear the Actigraph for 2 or more days, with a valid ‘day’ being defined as having a minimum of 10 h of wear time. These ten children did not differ from the greater sample in age, Fe status measures, inflammation, amount of *bhakri* consumed or any other variable tested (on the basis of one-way ANOVA, data not shown). Because these unusable data were distributed fairly evenly between the treatment groups and constituted < 10 % of the overall sample, they were not likely to introduce to bias in a particular direction.

Finally, because this analysis used the participants with the lowest Fe status at baseline, the results of this study (*n* 112) may be less generalisable than if we had been able to assess the full sample from the parent study (*n* 246). This analysis was conducted as part of an efficacy study to determine the potential for using Fe-biofortified pearl millet to improve Fe status, with PA as a secondary outcome, in Indian schoolchildren and the results should be interpreted accordingly. Future research should investigate whether consuming additional Fe through dietary change in real-world conditions, such as those in a large, population-wide effectiveness study, show similar results on LPA, sedentary behaviour and other aspects of life related to PA such as fitness, fatigue, cognition and general health.

### Conclusions

Understanding whether and how micronutrient deficiencies affect children’s PA patterns in relation to their health is a topic that needs further exploration. This study is one of the first to use objective measures of PA and micronutrient status in a randomised controlled trial and is the first to demonstrate the effects of consuming an Fe-biofortified staple food on PA in children. The results of this study show that consuming Fe-biofortified pearl millet compared with conventional pearl millet increased LPA by 22 min/d Indian schoolchildren. In addition, this relationship appeared to be dose-dependent, with children showing increased LPA and reduced sedentary behaviours as they consumed greater amounts of Fe from pearl millet each day. These changes could subsequently contribute to improved health indicators, healthier motor and cognitive development, and reduced risk of mortality in this population.
